# Chronic inflammation: key player and biomarker-set to predict and prevent cancer development and progression based on individualized patient profiles

**DOI:** 10.1007/s13167-019-00194-x

**Published:** 2019-11-20

**Authors:** Shehua Qian, Olga Golubnitschaja, Xianquan Zhan

**Affiliations:** 1 Key Laboratory of Cancer Proteomics of Chinese Ministry of Health, Xiangya Hospital, Central South University, 87 Xiangya Road, Changsha, 410008 Hunan People’s Republic of China; 2 Hunan Engineering Laboratory for Structural Biology and Drug Design, Xiangya Hospital, Central South University, 87 Xiangya Road, Changsha, 410008 Hunan People’s Republic of China; 3 State Local Joint Engineering Laboratory for Anticancer Drugs, Xiangya Hospital, Central South University, 87 Xiangya Road, Changsha, 410008 Hunan People’s Republic of China; 4 Radiological Clinic, UKB, Excellence Rheinische Friedrich-Wilhelms-University of Bonn, Sigmund-Freud-Str 25, 53105 Bonn, Germany; 5 Breast Cancer Research Centre, UKB, Excellence Rheinische Friedrich-Wilhelms-University of Bonn, Bonn, Germany; 6 Centre for Integrated Oncology, Cologne-Bonn, Excellence Rheinische Friedrich-Wilhelms-University of Bonn, Bonn, Germany; 7 Department of Oncology, Xiangya Hospital, Central South University, Changsha, 410008 Hunan People’s Republic of China; 8 National Clinical Research Center for Geriatric Disorders, Xiangya Hospital, Central South University, Changsha, 410008 Hunan People’s Republic of China

**Keywords:** Chronic inflammation, Cancer, Inflammatory factors, Biomarkers, Predictive preventive personalized medicine, Patient stratification, Individualized patient profile, Risk factors, Modifiable and preventable, Genetics, Epigenetics, Multiomics, Machine learning, Big data analysis, Global statistics, Collateral pathologies, Phenotyping

## Abstract

A strong relationship exists between tumor and inflammation, which is the hot point in cancer research. Inflammation can promote the occurrence and development of cancer by promoting blood vessel growth, cancer cell proliferation, and tumor invasiveness, negatively regulating immune response, and changing the efficacy of certain anti-tumor drugs. It has been demonstrated that there are a large number of inflammatory factors and inflammatory cells in the tumor microenvironment, and tumor-promoting immunity and anti-tumor immunity exist simultaneously in the tumor microenvironment. The typical relationship between chronic inflammation and tumor has been presented by the relationships between *Helicobacter pylori*, chronic gastritis, and gastric cancer; between smoking, development of chronic pneumonia, and lung cancer; and between hepatitis virus (mainly hepatitis virus B and C), development of chronic hepatitis, and liver cancer. The prevention of chronic inflammation is a factor that can prevent cancer, so it effectively inhibits or blocks the occurrence, development, and progression of the chronic inflammation process playing important roles in the prevention of cancer. Monitoring of the causes and inflammatory factors in chronic inflammation processes is a useful way to predict cancer and assess the efficiency of cancer prevention. Chronic inflammation-based biomarkers are useful tools to predict and prevent cancer.

## Introduction

In medicine, cancer refers to a malignant tumor originating from epithelial tissue and is the most common type of malignant tumor. Cancer has biological characteristics such as abnormal cell differentiation and proliferation, loss of growth control, invasiveness, and metastasis [1]. Cancer occurrence is a multifactor, multistep complex process [2], including carcinogenesis, cancer promotion, and evolution. Infection, occupational exposure, environmental pollution, unreasonable diet, and genetic factors are closely related to cancer. For example, in 2018, lung cancer remained the world’s leading malignant tumor in morbidity and mortality, followed by colorectal cancer, gastric cancer, and liver cancer [3]. The mortality rate of these cancers is high, but it is well known that there is currently no effective method for accurate diagnosis and treatment of these cancers. Therefore, it can only be caught by prevention.

Inflammation is a defensive response to stimulation, characterized by redness, swelling, heat, pain, and dysfunction. In general, short-term inflammation is beneficial and an automatic defense response of the human body, but sometimes inflammation is also harmful; for example, it attacks the body’s own tissue and there is inflammation in transparent tissue. The persistence of inflammation in the body might be transformed into chronic inflammation [4]. Chronic inflammation can cause diabetes, autoimmune diseases, neurodegenerative disease, and cancer [4–7]. Chronic inflammation leads to loss of tissue structure, excessive tissue remodeling, and modification of protein self-neutralizing DNA caused by oxidative stress, all of which increase the risk of cancer development [8, 9]. Many studies have shown that chronic inflammation is associated with cancer [10–12]. The factors causing chronic inflammation are physical factors such as low temperature and radiation, chemical factors, and biological factors such as viruses, bacteria, and fungi [13].

The in-depth study of cancer reveals a strong relationship between cancer and inflammation [14]. For example, gastric cancer is associated with chronic gastritis, lung cancer is associated with chronic inflammation of the lungs, nasopharyngeal carcinoma is associated with Epstein–Barr virus, cervical cancer is associated with cervicitis, and liver cancer is associated with chronic hepatitis [15, 16]. Many studies have shown that inflammatory cells can promote the occurrence and development of tumors [17, 18], because inflammatory cells can promote cancer cell proliferation, angiogenesis, and tumor invasion, and change the efficacy of certain anti-cancer drugs [19, 20]. An important reason for the high mortality rate of cancer is that it can metastasize in the body. Some studies have shown that inflammation can help cancer metastasize. Coffelt et al. found that γδ-T cells and neutrophils can promote lung and lymph node metastasis in breast cancer patients [21]. In addition, activated inflammatory cells, hyenas release reactive oxygen species, can promote tumor progression [11]. At the same time, cancer treatment can also trigger inflammatory reactions, and then cause trauma, necrosis, and tissue damage, that might stimulate tumor recurrence and resist treatment [11]. In the tumor microenvironment, tumor-promoting immunity and anti-tumor immunity exist simultaneously [22]. When the tumor promotes immunity, the tumor cells grow faster, and on the contrary, the tumor cells are cleared [23]. As you can see from the above, the relationship between cancer and inflammation is very close. Although the biological characteristics of cancer are distinctive, the mechanism of tumor occurrence and development is still unclear. It is not impossible to use inflammatory factors as markers for prediction, prevention, and early diagnosis of cancer in the future. After all, many inflammatory factors have been used as biomarkers for cancer prediction, recurrence, and prognosis; for example, interleukin-6 (IL-6), tumor necrosis factor-α (TNF-α), and Wnt-1 can predict the occurrence and recurrence of hepatocellular carcinoma [24], and chitinase 3-like 1 is a predictor of inflammatory tumors in lung cancer models [25] and several inflammation-based prognostic systems, including platelet counts, systemic inflammatory scores, and Glasgow outcome scores [26].

## Chronic inflammation in different types of cancers

Chronic inflammation is involved in every cancer. Here, various factors causing chronic inflammation associated with lung cancer, gastric cancer (GC), and liver cancer are taken as examples to address chronic inflammation in cancer.

### Smoke causing chronic inflammation associated with lung cancer

Lung cancer is the first most common type of cancer in men and women, and the first most common cause of cancer-related death [3]. It is one of the most malignant tumors that threat the health and life of the population. Lung cancer can generally be divided into small cell lung cancer and nonsmall cell lung cancer. Among them, nonsmall cell lung cancer accounts for about 80% of them [27]. Chronic obstructive pulmonary disease (COPD) is a chronic bronchitis and/or emphysema characterized by airflow obstruction that can further develop into a common chronic disease of pulmonary heart disease and respiratory failure. It is associated with abnormal inflammatory reactions of harmful gases and harmful particles and has high morbidity and mortality. More and more studies have found that the COPD that is a chronic inflammatory pulmonary disease is an important risk factor of lung cancer [16, 28–32]. COPD-induced hypoxia in the lungs activates hypoxic transcription factors that inhibit apoptosis, which might trigger lung cancer [33]. In patients with COPD, chronic inflammation leads to a decrease in the ability of the lungs to clear, and toxic substances are in contact with respiratory epithelial cells, making patients more likely to acquire lung cancer [34]. In addition, lung damage caused by chronic inflammation of COPD increases endogenous DNA damage, thereby increasing the risk of carcinogenesis [35].

Cigarette smoke is made up of more than 7000 different compounds, most of which have adverse effects on respiratory cells [36]. Cigarette smoke contains oxidants and toxic molecules [37]. Smoking plays an important role in lung cancer and COPD [38, 39]. Numerous studies have shown that the longer smoking time and the greater amount of smoking can cause the greater probability of developing lung cancer and the higher mortality rate of lung cancer [40–42]. Smoking can cause inflammation and oxidative stress in the lungs, which can trigger COPD and lung cancer [43]. Toxic gas molecules in cigarettes, such as reactive oxygen species and reactive nitrogen species, enter the lungs through the upper respiratory tract, causing oxidative stress in the lung, and sustained oxidative stress is a major cause of inflammation and COPD [44]. The reactive oxygen species can also directly lead to the damage of DNA and the degradation of tumor suppressors, and then might lead to carcinogenesis [45, 46]. Zuo et al. believed that oxidative stress caused by the cigarette smoke oxidant could activate nuclear transcription factor κB (NF-κB) and mitogen-activated protein kinase (MAPK), thereby promoting the release of chemokine and cytokines and then triggering an inflammatory response [47]. In addition, when cigarette smoke enters the lungs through the respiratory tract, the body’s first immune defense, innate immunity, is opened, resulting in reduced mucosal cilia clearance and excessive mucus secretion and infiltration of the airway wall by natural killer lymphocytes and macrophages [48]. Cigarette smoke can also be used as a foreign antigen to stimulate the body’s adaptive immune response, thereby activating some immune cells and triggering the release of inflammatory factors. Arnso et al. and Churg et al. believed that cigarette smoke promoted the release of TNF-α, IL-1, IL-6, and IL-8 [49, 50]. Since IL-6 and TNF-α can predict the occurrence and recurrence of hepatocellular carcinoma [24], TNF-α, IL-1, IL-6, and IL-8 might be used as biomarkers for prevention and prediction of lung cancer.

Except for smoking associated with lung cancer, the risk factors that lead to lung cancer include air pollution, genetic risk factors, occupational exposures, diet, and alcohol [47]. Lung cancer is a malignant tumor with high incidence and high mortality. Therefore, lung cancer can only be prevented from the source in advance, such as not smoking, not smoking second-hand smoke, maintaining good eating habits, and so on.

### *Helicobacter pylori* causing chronic inflammation associated with GC

In 2018, the mortality rate of GC was 8.2%, and GC is the third most lethal cancer among 36 cancers [3]. Although the mortality rate of GC is so high, specific early diagnosis symptoms have not been found [51–53]. GC is a malignant tumor originating from the gastric mucosal epithelium. According to Lauren’s classification, the GC can be divided into intestinal type GC and diffuse type GC [54]. Glandular cavity formation is common in intestinal type GC, and the adjacent mucosa is often accompanied by extensive atrophic gastritis and intestinal metaplasia. Intestinal GC is often thought to be secondary to chronic atrophic gastritis.

*Helicobacter pylori* (*H. pylori*) is a spiral, microanaerobic, gram-negative bacterium that is very demanding on growth conditions. In 1983, it was successfully isolated from gastric mucosal biopsies from patients with chronic active gastritis [55]. It is the only microbial species known to survive in the human stomach. *H. pylori* is generally acquired at a young age and generally lasts for a lifetime [56]. *H. pylori* causes acute and chronic gastritis, leading to progressive damage to the gastric mucosa. Therefore, it is associated with many important upper gastrointestinal diseases, including esophageal cancer, chronic gastritis, chronic ulcers, distal gastric adenocarcinoma, and gastric lymphoma [57–59]. Fortunately, only about 5% of infected people can acquire GC [60]. It is generally believed that the clinical process of *H. pylori* infection is such that *H. pylori* colonizes the gastric mucosa and settles infection, causing chronic, superficial gastritis after several weeks or months and develops into GC after several years or decades. It refers to intestinal ulcer, gastric ulcer, lymphoproliferative gastric lymphoma, chronic atrophic gastritis, etc., while the latter is the most dangerous factor leading to GC. Experts believe that *H. pylori* infection increases the risk of GC development by about 2 times [61]. *H. pylori* can cause GC in two ways. One of them is that virulence factors of *H. pylori* directly cause epithelial cell damage, leading to epithelial cell apoptosis and proliferation and the production of inflammatory factors; the other is that *H. pylori* can pass through gastric mucosal cells, triggering innate immunity and specific immunity, and the body secretes a variety of inflammatory factors [62, 63].

Except for host and environmental influence, *H. pylori* affects mucosal and systemic immune responses through bacterial virulence factors that affect cytokine secretion and recruitment of different inflammatory cells [64, 65]. So far, in *H. pylori*, the best identified bacterial virulence factors that are associated with inflammation and carcinogenesis are the vacuolating cytotoxin (VacA) and the cag type-IV secretion system (T4SS), and its translocated effecter protein, cytotoxin-associated gene A (CagA) [66]. The cytotoxin-associated gene pathogenicity island (Cag-PAI) can promote pathogenic virulence factors such as CagA into gastric epithelial cells through the T4SS [67]. After entering the gastric epithelial cells, the CagA undergoes tyrosine phosphorylation and then binds to tyrosine phosphatase SHP-2 which is Src homology-domain-containing protein tyrosine phosphatase [64]. Sustained activation of SHP-2 by CagA induces apoptosis of gastric epithelial cells and, therefore, might cause atrophic gastritis associated with *H. pylori* [68, 69]. Atrophic gastritis is a well-recognized precancerous lesion, and it might develop into GC [70, 71]. In addition, activated SHP-2 is able to induce MAPK signaling through Ras/Raf-dependent and Ras/Raf-independent mechanisms [72], and the MAPK cascade is a highly conserved module that is involved in various biological processes, including inflammation, proliferation, differentiation, and anti-apoptosis. The Cag-PAI also can directly activate nucleotide-binding oligomerization domain through the T4SS, and then activate NF-κB [72], causing DNA damage, ultimately leading to chemotatic and proinflammatory effects. The chemokines and inflammatory factors produced by chemotatic and proinflammatory effects might promote the development of chronic gastritis, which in turn triggers GC. Therefore, target drugs might be designed according to the epithelial cell signaling in *H. pylori* infection to prevent the formation of GC in patients with *H. pylori* infection, such as T4SS inhibitors, and Cag-PAI inhibitors.

The cell wall of *H. pylori* is a lipopolysaccharide (LPS), which is an endotoxin. When *H. pylori* enters the stomach, LPS is captured as an important antigen molecule by antigen-presenting cells and then causes a series of immune responses in the body, which in turn causes the body to secrete a variety of inflammatory factors. LPS can bind to the transmembrane recognition receptor toll-like receptor 4 (TLR4) and then activates the Toll-Like receptor signaling pathway. Pathogen recognition of TLRs causes rapid activation of innate immunity by inducing the production of proinflammatory cytokines and upregulation of costimulatory molecules. The TLR signaling pathway is divided into two groups: a myeloid differentiation factor 88 (MyD88)-dependent pathway, which leads to the production of proinflammatory cytokines that are rapidly activated by NF-κB and MAPK, and a MyD88-independent pathway associated with the induction of interferon-beta (IFN-β) and IFN-inducible genes, as well as the maturation of dendritic cells that are slowly activated by NF-κB and MAPK. These persistent inflammatory factors can cause DNA damage and chronic gastritis, which might eventually lead to GC. Of course, it is obviously to simplify the process of chronic gastritis induced by H. pylori and then induces gastric cancer, and the specific induction process needs to be further studied.

*H. pylori* can evade the body’s immune system for a long time, and this mechanism might cause cell damage and chronic inflammation [63]. Infection of the stomach by *H. pylori* can cause the body to produce reactive oxygen and nitrogen species, which causes DNA damage to trigger GC [73]. In addition, although the stomach environment is very acidic, *H. pylori* still has good colonization ability in the stomach [74]. In summary, *H. pylori* can colonize the stomach for a long time, and then cause chronic gastritis, which might eventually lead to the development of GC.

### Hepatitis B and C virus causing chronic inflammation associated with liver cancer

Liver cancer can be divided into primary and secondary liver cancer. Primary liver malignant tumors originate from the epithelial or mesenchymal tissues of the liver. The former is called primary liver cancer and is a highly harmful malignant tumor. The latter is called sarcoma and is rare compared with primary liver cancer. In 2018, liver cancer remained the world’s leading malignant tumor in morbidity and mortality [3]. Liver cancer is a malignant tumor with multiple carcinogenic factors which include alcohol, viral infections, congenital immune diseases, and genetic diseases [75, 76]. Among these carcinogenic factors, the host immune system and viral infection with hepatitis B virus (HBV) and hepatitis C virus (HCV) are the most important risk factors [77, 78]. It is common knowledge that persistent hepatitis can lead to liver cirrhosis and then liver cancer, which is called the liver cancer trilogy. Therefore, persistent hepatitis can be considered as the initial initiator. Chronic hepatitis is inseparable from HBV and HCV. This review will explain the relationship between HBV and HCV and chronic hepatitis and liver cancer.

#### HBV causing chronic inflammation associated with liver cancer

HBV is a hepadnavirus that belongs to the family of hepatic viruses, and was discovered in 1966 [79]. The complete HBV consists of the envelope that includes an envelope and a nucleocapsid whose core contains a double-stranded circular DNA [80]. The DNA is infectious. HBV adheres to the surface of hepatocytes through low-affinity receptors (such as heparan sulfate, proteoglycan, etc.) and then binds to the viral receptor through the pre-S1 region of the large envelope protein to mediate the endocytosis of the virus. The sodium ion-taurocholic acid transport peptide is an important receptor that mediates the entry of HBV into cells and establishes infection. The endocytic virus envelope and the membrane of the swallowed membrane release the capsid into the cytoplasm, and the capsid is transported. The viral genomic rcDNA to the interior of the nuclear pore complex is released into the nucleus. Within the nucleus, rcDNA may be converted to covalently closed circular DNA (cccDNA) by the DNA replication machinery of the cell. The cccDNA has high stability and can last for several months to several years in the nucleus, which is the root cause of viral rebound after antiviral treatment. Therefore, clearing cccDNA is a decisive significance for eradication of hepatitis B [81]. In 2016, approximately 260 million people were infected with HBV around the world [82].

HBV is transmitted through the blood, sexual intercourse, and vertical transmission [83]. For adults, once infected with HBV, the body with normal physiological function immediately responds and recovers, but it may develop chronic HBV infection in patients with liver dysfunction or impaired immune function [84]. Therefore, the chronic infection rate caused by HBV infection in infants and young children is as high as 90% [83]. During the infection, the body immediately started to protect itself from the systemic epidemic [85], although the immune response caused by HBV and its role in the mechanism of chronic hepatitis B is not clear [86]. Chronic hepatitis B can cause liver damage, leading to liver fibrosis, cirrhosis, and even liver cancer [87]. HBV-infected liver cytokine family binds to its corresponding receptor to activate the Janus kinase–signal transducer and activator of transcription (JAK–STAT) signaling pathway, causing DNA damage, thereby promoting hepatocyte proliferation, inhibiting hepatocyte apoptosis, and then triggering liver cancer [88]. He et al. also believed that NF-κB can promote the occurrence of liver cancer through HBV infection of liver cells and liver inflammation [88]. In addition, studies have shown that patients with chronic HBV infection have higher levels of lipopolysaccharide in the blood [84]. This might suggest that there may be activation of Toll-like receptor pathway in chronic HBV-infected individuals. The relationship between the TLR pathway and tumors has been briefly described in the gastric cancer section.

#### HCV causing chronic inflammation associated with liver cancer

HCV is a single-stranded positive-strand RNA virus, which is surrounded by a lipid-containing capsule in the nucleocapsid and has a condyle on the capsule. HCV is classified in the Flaviviridae family. HCV often infects the liver, causing chronic HCV infection, which then triggers hepatitis, cirrhosis, liver cancer, and more [89]. Most patients with chronic HCV infection develop chronic hepatitis C [90, 91]. Li et al. believed that the mechanism by which HCV develops into chronic hepatitis C is that HCV RNA can activate NF-κB and cellular inflammatory factors (such as TNF, IL-6, etc.) through TLRs, and these cellular inflammatory factors interact with their receptors to activate the JAK–STAT, MAPK, and phosphatidylinositol 3-kinase–AKT (PI3K–AKT) signaling pathways [92]. These signaling pathways are closely related to the formation of tumors [93–95]. These cellular inflammatory factors can also provide a tumor microenvironment for the development and progression of liver cancer [96]. Moussa et al. found that the immunopositive rate of pSmad2/3 and Smad4, which are the cellular inflammatory factors, increased with the degree of chronic hepatitis C, liver fibrosis, and liver cancer by the study on patients with chronic hepatitis C, liver cancer patients, and normal controls [97]. The reason why HCV can cause chronic HVC infection is because the HCV can evade the body’s monitoring of innate and adaptive immunity [98]. HCV can evade the body’s immune surveillance through a variety of mechanisms, including changing the differentiation direction of CD4 cells, allowing them to differentiate into TH2 and TH17 cells, which destroy the function of natural killer cells [99].

In addition, studies have shown that HCV can also cause liver cancer by itself. They believe that HCV core protein is an important factor in HCV-induced liver disease [100]. This protein is closely related to cell apoptosis, growth, proliferation, etc. and is related to the regulation of signaling pathways such as the MAPK pathway and cyclooxygenase-2 [101]. Abnormalities of these signaling pathways are closely related to liver cancer [102]. The viral protein of HCV can cause oxidative stress in the liver, and oxidative stress can promote the occurrence of liver cancer [103].

Except for HBV and HCV, diet, alcohol, and genetic risk factors are also the predisposing factors for liver cancer [104]. The clinical symptoms of early onset of liver cancer are not obvious, so many patients diagnosed with liver cancer are from the middle and late stages of the rod. Therefore, prevention of liver cancer from the source is extremely important. For HBV and HCV, it is necessary to prevent its spread through the blood. Good habits and optimism are also important for other risk factors for liver cancer. In addition, both HBV- and HCV-induced liver cancer are associated with activation of signaling pathways, so target drugs can be designed to prevent the development of chronic hepatitis patients into liver cancer patients.

## Proinflammatory factors and inflammatory factors in chronic inflammation process in cancer

Chronic inflammation is a very complex process. Except for initiating factors causing chronic inflammation, including physical factors such as low temperature and radiation, chemical factors such as different chemical carcinogens, and biological factors such as viruses, bacteria, and fungi, there are lots of proinflammatory factors and inflammatory factors that are secreted by leukocytes and mast cells involved in the chronic inflammation processes, including IL-6, IL-1, NF-κB, TNF-α, STAT3, tumor growth factor-beta (TGF-β), and so on. And these factors promote proliferation and differentiation of epithelial cells and endothelial cells [105], then might induce cancers. Lee et al. found TNF-like weak inducer induced inflammation with real-time polymerase chain reaction (RT-PCR), western blotting, and enzyme-linked immunosorbent assay (ELISA) [106]. Khodabandehlon et al. found the existence of human papillomavirus was related to tumor progress and the increase of inflammatory cytokines (IL-6, IL-17, IL-1, NF-κB, TNF-α, and TGF-β) with ELISA and RT-PCR [107]. Many proinflammatory factors and inflammatory factors are associated with chronic inflammation and cancer. Here, IL-1, IL-6, and IL-8 are taken for example for detailed discussions.

### IL-1 associated with cancer

IL-1 is a cytokine of the chemokine family and is produced primarily by macrophages. So far, the IL-1 family has 11 members, but the most important ones are IL-1α and IL-1β. The most studied subtype of IL-1 is IL-1β [108].

IL-1β is mainly produced by tissue macrophages, skin dendritic cells, and blood mononuclear cells. The biological activity of the IL-1β precursor is biologically active after being subjected to enzymatic treatment and is mainly present in the microcirculation system [109]. Under normal physiological conditions, IL-1 has an anti-tumor effect, but when the body is in persistent chronic inflammation, IL-1 has a tumor-promoting effect, at which time IL-1β supports tumor development [110, 111]. IL-1β activates vascular endothelial growth factor, which promotes blood vessel growth and provides nutrients for tumor growth [112, 113]. At the same time, IL-1β can also induce chronic inflammation and activate blood endothelial cells, thereby promoting the metastasis and invasiveness of cancer cells [114]. IL-1β activates the NF-κB signaling pathway of myeloid cell lines (MDSCs), and NF-κB is an important link between inflammation and cancer. MDSCs can secrete IL-6 and TNF-α, which can promote tumor growth [115]. When IL-1 β binds to its corresponding receptors, it can activate MyD88 and IL-1 receptor–associated kinase-4 (IRAK4), which leads to the phosphorylation of IRAK2 and IRAK1 and then activates NF-κB. Activated NF-κB can enter the nucleus and promote the transcription of some inflammatory genes [116]. It leads to the increase of the level of inflammatory factors in the body, which may promote the occurrence of tumor.

IL-1 is involved in the angiogenesis and proliferation of cancer cells, which may promote the development of cancer [117, 118]. IL-1 also can promote the expression of vascular cell adhesion factor-1, thereby promoting adhesion and metastasis of cancer cells [119]. Han et al. found that IL-1 is involved in the invasion of gastric cancer with a mice model [120]. In addition, several studies have shown that IL-1β is involved in the development of gastric cancer [121, 122]. The study of IL-1α is few, but it is also involved in tumor progression and metastasis, which can activate NF-κB and promote tumor growth [123].

In conclusion, the proinflammatory cytokine IL-1 is closely related to the occurrence, development, metastasis, and invasion of tumors. Therefore, IL-1 may be used as a biomarker for tumor diagnosis and prognosis in the future.

### IL-6 associated with cancer

IL-6 is a cytokine of the chemokine family. IL-6 is a protein of 184 amino acids with a molecular weight of 21–28 kDa, and it was found in 1968 [124]. IL-6 is mainly produced by macrophages, T lymphocytes, B lymphocytes, monocytes, and so on [125]. IL-6 is a multifunctional cytokine that promotes tumor cell proliferation, invasion, and metastasis; inhibits tumor cell apoptosis; and promotes blood vessel growth [126]. IL-6 mainly relies on the activation of multiple signaling pathways to participate in the development of tumors, such as the JAK2/STAT3 signaling pathway, PI3K/AKT signaling pathway, RAS/MAPK signaling pathway, and so on [127]. Upon binding of IL-6 to the IL-6 receptor (IL-6R), GP130 is activated to form a dimer, which induces phosphorylation of JAK1 and JAK2 leading to phosphorylation of STAT1 and STAT3. Phosphorylated STAT3 can enter the nucleus and induce a variety of gene transcription, such as cFOX, IRF-1, Bcl2, etc., which are involved in cell growth, differentiation, inhibition of apoptosis, and promotion of vascular production and cell adhesion [124, 128–130]. STAT3 can also induce oncogenes that are associated with cell proliferation and metastasis. In addition, IL-6 binds to IL-6R to activate the PI3K–AKT signaling pathway, which induces phosphorylation of JAK and PI3K and activates AKT, to regulate several genes involved in cell survival [131].

In conclusion, IL-6 is involved in the proliferation, metabolism, metastasis, invasion, and angiogenesis of various tumors and has been found to have high expression of IL-6 in various tumors, such as breast cancer, colorectal cancer, prostate cancer, lung cancer, ovarian cancer, and so on. Moreover, Xu et al. thought that serum IL-6 might be a potential biomarker for colorectal cancer [132]. And inhibitors of IL-6, IL-6R, GP130, JAK, and STAT3 may be targets for tumor therapy in the future.

### IL-8 associated with cancer

IL-8 is a cytokine of the chemokine family. IL-8 is mainly produced by endothelial cells, epithelial cells, fibroblasts, etc., and its active form is composed of 69, 72, 77, and 79 amino acids, respectively, and has a molecular weight of about 8 kDa [133]. The receptors for IL-8 are CXCR1 and CXCR2. IL-8 generally only undergoes biological function when it binds to a receptor. For example, Yung et al. and other studies found that IL-8 binds to CXCR2 and activates transforming factor-β-activating enzyme 1 (TAK1)/NF-κB signaling, which in turn increases the invasiveness of ovarian cancer cells [134]. Sharma et al. found that IL-8 binds to CXCR1/CXCR2 and indirectly promotes angiogenesis, proliferation, and invasion of cancer cells and promotes the progression of glioblastoma multiforme, and the level of IL-8 is higher and the patient’s prognosis is worse [135]. In addition, Zheng et al. found that M2 macrophages produced IL-8, which activated STAT3 and phosphorylated it, leading to increased expression of lung adenocarcinoma transcript-1 that was associated with lung adenocarcinomas metastasis [136]. IL-8 can induce PI3K phosphorylation and then activate AKT phosphorylation, which in turn increases blood vessel growth, cancer cell survival, and migration [137]. IL-8 also regulates cell proliferation, survival, and invasion by activating MAPK and ERK1/2 phosphorylation [137]. In brief, IL-8 is highly expressed in a variety of cancers, such as colon cancer, lung cancer, breast cancer, and so on [138]. And IL-8 is closely related to tumor growth, invasion, and metastasis [139–141].

**Fig. 1:**
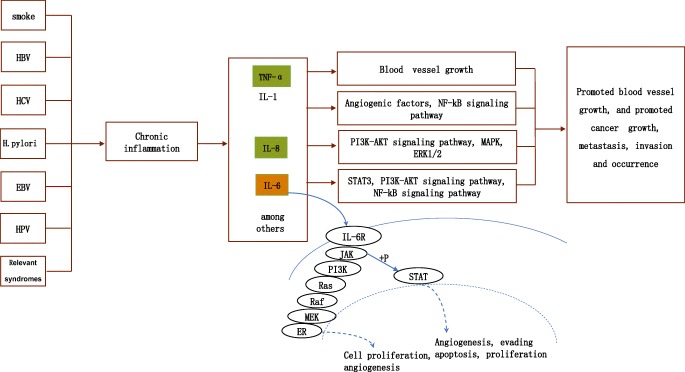
A molecular model proposed for chronic inflammation in cancer. The orange box means the potential therapeutic targets and biomarkers for cancer. The green box means potential therapeutic targets for cancer. The corresponding information on relevant syndromes is provided in the article, for example, regarding the “Flammer syndrome” phenotype, “Individualized patient profiling is instrumental for cancer prediction and prevention” section

Besides IL-1, IL-6, and IL-8, other proinflammatory factors are also associated with the occurrence and development of cancer, such as TNF-α, TGF-β, BCA-1, and so on (Fig. 1). These proinflammatory factors generally promote the development of cancer by activating signaling pathways (Table 1). Although TGF-β has an inhibitory effect on the cancer cell cycle in the early stage of tumors, it also has a tumor-promoting effect [142]. TNF-α promotes blood vessel growth and promotes tumor progression and metastasis [142], and TNF-α can be used to predict the occurrence and recurrence of liver cancer [24]. In addition, Rossi et al. found that TNF-α could increase the invasiveness of melanoma cells [143], and Tan et al. found that TNF-α could be a potential therapeutic target for hepatocellular carcinoma [144]. NF-κB is an important immune protein and NF-κB signaling pathway is closely related to the development and progress of human tumor [145]. NF-κB has been shown to be a linker of inflammation and cancer in mouse models [146]. A variety of cytokines can activate NF-κB signaling pathway. Most proinflammatory factors activate STAT3, and chitinase 3-like 1 that is a downstream gene of the STAT3 signaling pathway is a potential biomarker to predict inflammatory lung cancer in a STAT3-induced mouse lung cancer model [25]. In the IL-6 family, in addition to IL-6 associated with inflammatory tumors, both IL-23 and IL-11 are associated with tumors. For example, Wang et al. found that IL-23 promotes the development of gastritis, which might lead to gastric cancer [147], and Li et al. found that IL-23 can also promote the metastasis of liver cancer [148]. Yang et al. [149] and Lay et al. [150] found that IL-11 is involved in the development of esophageal squamous cell carcinoma and endometrial cancer. In addition, some scientists have found that IL-17A can promote the formation of tumor blood vessels [151, 152], thereby contributing to the proliferation and invasion of tumor cells. IL-5 promotes the metastasis and invasion of lung cancer and bladder cancer cells [153, 154]. Although IL-33 can inhibit the growth of lung adenocarcinoma [161], it is also involved in the invasion and migration of glioma cells [155], promotes the proliferation of colorectal cancer cells [156], and promotes the role of ovarian cancer [157]. Similar to IL-33, IL-15 is also a controversial cytokine that most researchers have found to have anticancer activity, but Gupta et al. found that it promoted colonal expansion of B cell chronic lymphocytic leukemia [158]. Besides cytokines, chemokines are also involved in the development of inflammatory tumors. CCL11 played an important role in the proliferation and invasion of ovarian cancer cells [159] and was involved in the progression of glioblastoma [160]. Therefore, since so many inflammatory and proinflammatory factors are associated with tumors, whether these factors can be used as biomarkers for specific tumor prediction, prevention, and prognosis is worthy of further study.

**Table 1:** Mechanisms of IL-1, IL-6, IL-8, and other proinflammatory factors or downstream molecules of signaling pathway which are activated by proinflammatory factors in different cancers

Proinflammatory factors or downstream molecules of signaling pathways which are activated by proinflammatory factors	Related cancer	Research models/species	Mechanisms	Biomarker to predict, prevent, or diagnose cancer or therapeutic target	Reference
IL-1	Lung cancer	Mouse	Induced angiogenic factors and promoted tumor growth	/	[113]
	Melanoma	B16 melanoma cells	Activated vascular endothelial growth factor and promoted tumor growth	/	[112]
	Melanoma	Mice	Induced endothelial factor and vascular adhesion factor and promoted the metastasis and invasion of cancer cells	/	[114]
	Gastric cancer	Mice	Activated NF-κB signaling pathway of MDSCs and promoted tumor growth	/	[115]
	Breast cancer	Human	Activated MyD88 and IRAK4, activated NF-κB, and promoted the metastasis of cancer cells	Predicted breast cancer patients at increased risk for developing bone metastasis	[116]
	Breast cancer	Mouse mammary cancer 4T1 cells	Promoted angiogenesis and proliferation of cancer cells	/	[118]
	Melanoma	Mice	Induced vascular cell adhesion molecule-1 and promoted melanoma metastasis	/	[119]
	Gastric cancer	Mice	Induced microRNA 135b and promoted metastasis of gastric cancer cells	/	[120]
	Gastric cancer	Mouse	Affected inflammatory and epithelial cells and enhanced mouse gastric carcinogenesis	/	[122]
IL-6	Renal cell cancer	/	Activated STAT3 and promoted growth, proliferation, and metastasis of cancer cells	/	[124]
	Colorectal cancer	Human	Activated STAT3 and promoted differentiation, proliferation, and survival of cancer cells	/	[130]
	Breast cancer	Human	Activated PI3K–AKT signaling pathway and regulated survival of cancer cells	A potential therapeutic target	[131]
	Colorectal cancer	Human	Activated NF-κB signaling pathway and promoted the occurrence of colorectal cancer	A potential biomarker for colorectal cancer	[132]
IL-8	Ovarian cancer	Human ovarian cancer cell lines	Activated TAK1/NF-κB signaling pathway and increased the invasion of ovarian cancer cells	A potential therapeutic target	[134]
	Glioblastoma multiforme	Glioblastoma multiforme cell lines	Bond to CXCR1/2 and promoted proliferation and invasion of tumor cells	/	[135]
	Lung adenocarcinoma	Cell lines	Activated STAT3 and promoted metastasis of tumor	/	[136]
	Pancreatic cancer	Cell lines	Activated PI3K/AKT signaling pathway and increased blood vessel growth, cancer cell survival, and migration	/	[137]
	Colon and lung cancer	Cancer cells	Activated MAPK and ERK1/2 phosphorylation and promoted proliferation, survival, and invasion of cancer cells	/	[138]
	Hepatocarcinoma	Human hepatocarcinoma cells	Activated AKT and promoted metastasis and growth of cancer cells	/	[139]
	Osteosarcoma	Cancer cells	Activated AKT and FAK and promoted metastasis, invasion, and proliferation of cancer cells	/	[141]
TNF-α	Breast cancer	/	Promoted blood vessel growth and promoted tumor growth and metastasis	/	[142]
	Hepatocellular carcinoma	Human	/	A potential biomarker for predicting the occurrence and recurrence of hepatocellular carcinoma	[143]
	Melanoma	Cell lines	Promoted cancer cells metastasis	/	[144]
	Hepatocellular carcinoma	Cell lines, human tissue, and mice	/	A potential therapeutic target	[145]
NF-κB	/	/	Regulated proliferation, survival and growth of cells, and linked between inflammatory and cancer	/	[146]
Chitinase 3-like 1	Lung cancer	Mouse	Downstream gene of STAT3	A potential biomarker for predicting inflammatory lung cancer	[25]
IL-23	Gastric cancer	Cell	Promoted gastritis and high expression of p53	/	[147]
	Hepatocellular	Human	Promoted hepatocellular carcinoma metastasis	/	[148]
IL-11	Esophageal squamous cell cancer	Cell lines	Promoted esophageal squamous cell cancer invasion and proliferation	/	[149]
	Endometrial cancer	Cell lines	/	A potential therapeutic target	[150]
IL-17	Lung cancer	Cell	Promoted tumor angiogenesis	A potential therapeutic target	[151]
	Cervical cancer	Human	Promoted angiogenesis and promoted cancer cells proliferation and invasion	A potential therapeutic target and diagnostic marker	[152]
IL-5	Lung cancer	Mouse	Promoted cancer cell metastasis	/	[153]
	Bladder cancer	Cell lines	Promoted the migration and invasion of bladder cancer cells	A potential therapeutic target	[154]
IL-33	Glioma	Human and cell lines	Involved in migration and invasion of cancer cells	A potential therapeutic target	[155]
	Colorectal cancer	Cell lines and mice	Promoted the proliferation of cancer cells	A potential therapeutic target	[156]
	Ovarian cancer	Cell lines	Promoted the development of cancer	A potential therapeutic target	[157]
IL-15	B cell chronic lymphocytic leukemia	Cells	/	/	[158]
CCL11	Ovarian cancer	Cells	Played an important role in the proliferation and invasion of ovarian cells	A potential therapeutic target	[159]
	Glioblastoma	Human	Involved in the progression of glioblastoma	A potential therapeutic target and diagnostic marker	[160]

## Multiomics and molecular networks in chronic inflammation process in cancer

Multiomics includes genomics, transcriptomics, proteomics, metabolomics, and radiomics [1], which is more and more widely used in clinical treatment and basic research of cancer [2, 162–165].

Genomics is a science of genomic mapping (including genetic maps, physical maps, transcriptional maps), nucleotide sequence analysis, gene mapping, and gene function analysis of all genes. Genomic information is increasingly being used in the diagnosis, treatment, and prognosis of tumors [166]. Gene mutations might occur during inflammation-induced cancer, and genomic techniques can be used to quickly understand the cause and then adopt an effective treatment.

Transcriptomics is a discipline that studies the transcription of genes and transcriptional regulation in cells at an overall level. The transcriptome is an important objective to study the development of cell phenotype and function and response diseases. One of the common features of tumors is splicing abnormality [1]. Therefore, the use of transcriptomics-related techniques to detect abnormal shear in the body is conducive to early detection of cancer, early treatment of cancer, and relief of patient suffering and economic burden.

Proteomics is a science in which the proteome is the research object to study the protein composition of cells, tissues, or organisms and their changes. Proteins might form protein variants due to post-translational modifications, cleavage, etc. [167], so the number of proteins is far more than the number of genes. Proteomics research mainly includes the extraction, separation, and identification of proteins. The methods for studying protein separation are mainly one-dimensional gel electrophoresis and two-dimensional gel electrophoresis [168], and the identification method is mainly mass spectrometry. It can be said that proteomics study is more important than genomics and transcriptomics study [169]. Proteomics studies attempt to compare the similarities and differences of protein expressions in different physiological or pathological conditions, and classify and identify related proteins. Therefore, it is extremely clinically meaningful to compare protein expressions in cancer patients and normal humans. It can provide a clinical basis for the diagnosis, treatment, and prognosis of cancer patients.

Metabolomics is a way to quantitatively analyze all metabolites in organisms, and to find out the relative relationship between metabolites and physiological and pathological changes. Most of the subjects are small molecular substances with relative molecular weight less than 1000. Metabolism is one of the indispensable life activities in the body. Many intracellular life activities take place at the metabolite level, such as cell signal release (cell signaling), energy transfer, and cell-to-cell communication which are regulated by metabolites. Cancer caused by inflammation leads to changes in the pathophysiological process of the body and eventually causes corresponding changes in metabolites. Through the analysis of some metabolites, and compared with the metabolites of normal people, it may be possible to find biomarkers for cancer and provide better methods for early diagnosis.

Radiomics is to obtain images of patients’ lesions by using some medical instruments, and then to further diagnose, predict, and analyze the massive image data to assist doctors in making the most accurate diagnosis. For example, CT, PET/CT, and MRI are used to diagnose the size of the tumor and the growth of the tumor to determine the benign and malignant tumors. During the occurrence and development of cancer that is induced by chronic inflammation, some signaling pathways are involved in this process, for example, MAPK signaling pathway [101], Toll-like receptor signaling pathway, NF-κB signaling pathway [145], JAK–STAT signaling pathway [88], and PI3K–AKT signaling pathway [92]. When chronic inflammation induces tumorigenesis and development, some genes, proteins, and metabolites in these signaling pathways might be abnormally expressed, so it may be possible to use these genes, proteins, and metabolites as biomarkers for tumor prevention and prediction and even as a drug target for clinical therapy.

Those multiomics and molecular network approaches promote one to consider chronic inflammation in cancer from a multiparameter systematic strategy angle, but not from a single one-parameter model in order to understand thoroughly the molecular mechanisms of chronic inflammation in cancer and discover more reliable and effective biomarkers targeting the chronic inflammation process associated with cancer to predict the occurrence and development of cancer, effectively prevent cancer, and even design reasonable assessment index to assess the effects of cancer prevention.

## Impaired wound healing and chronic inflammation as a clue to aggressive cancer development and progression

Wound healing refers to a highly complex repair process in which the body is subjected to external forces and the tissues such as the skin are broken or defective. It is mainly divided into the following four stages: hemostasis, inflammation, proliferation, and tissue remodeling [162]. The time required for wound healing is mainly related to the severity of the wound and the health of the body. Some wounds may only take a few days to heal. Some wounds may take years or decades to heal, but some wounds may never be able to heal, such as a tumor [163]. Inflammation is involved in the process of wound healing, and the slow healing of the wound may be related to the excessive and prolonged period of inflammation [163]. As can be seen from the foregoing, chronic inflammation can cause cancer. Therefore, it can be considered that impaired wound healing can promote the development and progression of cancer. At the same time, cancer can inhibit the healing of wounds, such as due to the use of radiotherapy and chemotherapy drugs, malnutrition in cancer patients, and abnormal immune system in cancer patients [163, 164].

## Individualized patient profiling is instrumental for cancer prediction and prevention

It is well known that there are various factors for cancer, including genetic factors, immune factors, and habit factors. The prediction and prevention of cancer need choosing the right solutions for the cause. Individualized patient profiling is a right solution, which can understand individual details, including detailed basic information, family history, and medical history. For example, Golubnitschaja et al. have made a case study on Flammer syndrome, which understands the patient’s illness and family history, and finally made recommendations based on predictive, preventive, and personalize medicine [165]. Kunin et al. [170] and Goncharenko et al. [171] have conducted an individualized patient profile analysis of dry mouth and Flammer syndrome (FS), and vaginal dryness in the form of questionnaires, respectively. This is also a good solution for cancer prediction.

## From phenotype to predictive biomarker panels: the “road map” for implementation

As can be seen from the foregoing, chronic inflammation can promote the occurrence and development of cancer. The underlying cause of chronic inflammation is the persistence of inflammatory factors and tissue damage. There are many kinds of tissue damage, such as wounds. Wound healing is generally divided into four phases: hemostasis, inflammation, proliferation, and remodeling. In the early stage of the wound, the vascular rupture is in a state of transient hypoxia. The hypoxia-inducing factor produced by this process plays an important role in the stages of angiogenesis, tissue remodeling, and inflammation, which can be used to judge the healing of the wound [172]. In addition, impaired wound healing is associated with FS [173], and FS-related systemic hypoxia can promote cancer metastasis, such as breast cancer [174].

## Conclusion and expert recommendation

The early clinical symptoms of cancer are not obvious and the social medical mechanisms in developing countries are not perfect, both of which lead to cancer in the middle and late stages of diagnosis. This brings more economic burdens to the patient’s family and brings more pains to the patient and a lower quality of life for the patient. Therefore, it is extremely important to find clinical diagnostic markers for early cancer. All cancers including GC, liver cancer, and lung cancer are also associated with inflammation. Although the molecular mechanism between cancer and chronic inflammation is still unclear, there is an inextricable link between cancer and chronic inflammation. It is of great significance for the study of biomarkers for cancer prevention and prediction that find how chronic inflammation induces cancer, develops cancer, and whether chronic inflammation is produced autonomously or is caused by acute inflammation. Of course, these tips allow us to continue to study the mechanisms between chronic inflammation and the development of cancer to discover inflammatory factors, cytokines, or key factors that regulate chronic inflammatory responses that may be involved in the development of cancer. This will alleviate the patient’s illness and bring the gospel to the patient and his family. In addition, FS has also been shown to be associated with the development of cancer and aggressive metastatic disease [172]. Therefore, for FS phenotype individuals, we should pay close attention to the state of the body to avoid cancer. Besides FS, diabetes, autoimmune diseases, and obesity are all inducing factors for cancer.

We recommend to emphasize and strengthen studies of chronic inflammation in cancers. Chronic inflammation in cancer involves many complex factors, complex processes, and multiple targets associated with cancers. It is necessary to use multiomics strategy in the study of chronic inflammation and cancers [1, 2, 162–165] to understand deeply the molecular mechanisms of chronic inflammation in cancer and discover more reliable and effective biomarkers targeting the chronic inflammation process associated with cancer to predict the occurrence and development of cancer, effectively prevent cancer, and even design reasonable assessment index to assess the effects of cancer prevention. Here, we propose the chronic inflammation-based strategy of cancer treatment: (i) reduce, inhibit, or eliminate the factors to cause chronic inflammation, including physical factors such as low temperature and radiation; chemical factors such as all types of chemical carcinogens; and biological factors such as viruses, bacteria, and fungi; (ii) strengthen the multiomics study of chronic inflammation to clarify the complex pathway networks of chronic inflammation, and establish biomarkers based on chronic inflammation pathway networks; and (iii) extensively study proinflammatory factor/inflammatory factor networks to identify inflammatory factor biomarkers and therapeutic targets to predict cancer, and inhibit or block the chronic inflammation progression to prevent occurrence and development of cancer.

## Abbreviations

*CagA*: Cytotoxin-associated gene A
*Cag-PAI*: Cytotoxin-associated gene pathogenicity island
*cccDNA*: Covalently closed circular DNA
*COPD*: Chronic obstructive pulmonary disease
*FS*: Flammer syndrome
*GC*: Gastric cancer
*H. pylori*: *Helicobacter pylori*
*HBV*: Hepatitis B virus
*HCV*: Hepatitis C virus
*IFN-β*: Interferon-beta
*IL-6*: Interleukin-6
*IL-6R*: IL-6 receptor
*IRAK4*: Interleukin-1 receptor–associated kinase-4
*JAK–STAT*: Janus kinase–signal transducer and activator of transcription
*LPS*: Lipopolysaccharide
*MAPK*: Mitogen-activated protein kinase
*MDSCs*: Myeloid cell lines
*MyD88*: Myeloid differentiation factor 88
*NF-κB*: Nuclear transcription factor κB
*PI3K–AKT*: Phosphatidylinosito 3-kinase–AKT
*T4SS*: Type-IV secretion system
*T4SS*: Type-IV secretion system
*TAK1*: Transforming factor-β-activating enzyme 1
*TGF-β*: Tumor growth factor-beta
*TLR4*: Toll-like receptor 4
*TNF-α*: Tumor necrosis factor-α
*VacA*: Vacuolating cytotoxin

## References

[1] Lu M, Zhan X (2018). The crucial role of multiomic approach in cancer research and clinically relevant outcomes. EPMA J.

[2] Cheng T, Zhan X (2017). Pattern recognition for predictive, preventive, and personalized medicine in cancer. EPMA J.

[3] Bray F, Ferlay J, Soerjomataram I, Siegel RL, Torre LA, Jemal A (2018). Global cancer statistics 2018: GLOBOCAN estimates of incidence and mortality worldwide for 36 cancers in 185 countries. CA Cancer J Clin.

[4] de Lavor EM, Fernandes AWC, de Andrade Teles RB, Leal A, de Oliveira Junior RG (2018). Essential oils and their major compounds in the treatment of chronic inflammation: a review of antioxidant potential in preclinical studies and molecular mechanisms. Oxidative Med Cell Longev.

[5] Naidoo V, Naidoo M, Ghai M (2018). Cell- and tissue-specific epigenetic changes associated with chronic inflammation in insulin resistance and type 2 diabetes mellitus. Scand J Immunol.

[6] Vendramini-Costa DB, Carvalho JE (2012). Molecular link mechanisms between inflammation and cancer. Curr Pharm Des.

[7] Aggarwal BB, Shishodia S, Sandur SK, Pandey MK, Sethi G (2006). Inflammation and cancer: how hot is the link?. Biochem Pharmacol.

[8] de Visser KE, Eichten A, Coussens LM (2006). Paradoxical roles of the immune system during cancer development. Nat Rev Cancer.

[9] Hardbower DM, Peek RM, Wilson KT (2014). At the bench: Helicobacter pylori, dysregulated host responses, DNA damage, and gastric cancer. J Leukoc Biol.

[10] Wu Y, Antony S, Meitzler JL, Doroshow JH (2014). Molecular mechanisms underlying chronic inflammation-associated cancers. Cancer Lett.

[11] Grivennikov SI, Greten FR, Karin M (2010). Immunity, inflammation, and cancer. Cell..

[12] Grivennikov SI, Karin M (2010). Inflammation and oncogenesis: a vicious connection. Curr Opin Genet Dev.

[13] Bartsch H, Nair J (2006). Chronic inflammation and oxidative stress in the genesis and perpetuation of cancer: role of lipid peroxidation, DNA damage, and repair. Langenbeck's Arch Surg.

[14] Lin WW, Karin M (2007). A cytokine-mediated link between innate immunity, inflammation, and cancer. J Clin Invest.

[15] Dobrovolskaia MA, Kozlov SV (2005). Inflammation and cancer: when NF-kappaB amalgamates the perilous partnership. Curr Cancer Drug Targets.

[16] Zhu HX, Shi L, Zhang Y, Zhu YC, Bai CX, Wang XD (2017). Myocyte enhancer factor 2D provides a cross-talk between chronic inflammation and lung cancer. J Transl Med.

[17] Coussens LM, Werb Z (2002). Inflammation and cancer. Nature..

[18] Balkwill F, Charles KA, Mantovani A (2005). Smoldering and polarized inflammation in the initiation and promotion of malignant disease. Cancer Cell.

[19] Hanahan D, Weinberg RA (2011). Hallmarks of cancer: the next generation. Cell..

[20] Neagu M, Constantin C, Caruntu C, Dumitru C, Surcel M, Zurac S (2019). Inflammation: a key process in skin tumorigenesis. Oncol Lett.

[21] Coffelt SB, Kersten K, Doornebal CW, Weiden J, Vrijland K, Hau CS (2015). IL-17-producing gammadelta T cells and neutrophils conspire to promote breast cancer metastasis. Nature..

[22] Ben-Baruch A (2006). Inflammation-associated immune suppression in cancer: the roles played by cytokines, chemokines and additional mediators. Semin Cancer Biol.

[23] Hadden JW (2003). Immunodeficiency and cancer: prospects for correction. Int Immunopharmacol.

[24] Han YF, Zhao J, Ma LY, Yin JH, Chang WJ, Zhang HW (2011). Factors predicting occurrence and prognosis of hepatitis-B-virus-related hepatocellular carcinoma. World J Gastroenterol.

[25] Yan C, Ding X, Wu L, Yu M, Qu P, Du H (2013). Stat3 downstream gene product chitinase 3-like 1 is a potential biomarker of inflammation-induced lung cancer in multiple mouse lung tumor models and humans. PLoS One.

[26] Song X, Zhu H, Pei Q, Tan F, Li C, Zhou Z (2017). Significance of inflammation-based indices in the prognosis of patients with non-metastatic colorectal cancer. Oncotarget..

[27] Reck M, Heigener DF, Mok T, Soria JC, Rabe KF (2013). Management of non-small-cell lung cancer: recent developments. Lancet (London, England).

[28] Sanchez-Salcedo P, Berto J, de-Torres JP, Campo A, Alcaide AB, Bastarrika G (2015). Lung cancer screening: fourteen year experience of the Pamplona early detection program (P-IELCAP). Arch Bronconeumol.

[29] Mannino DM, Aguayo SM, Petty TL, Redd SC (2003). Low lung function and incident lung cancer in the United States: data From the First National Health and Nutrition Examination Survey follow-up. Arch Intern Med.

[30] Punturieri A, Szabo E, Croxton TL, Shapiro SD, Dubinett SM (2009). Lung cancer and chronic obstructive pulmonary disease: needs and opportunities for integrated research. J Natl Cancer Inst.

[31] Omote N, Hashimoto N, Morise M, Sakamoto K, Miyazaki S, Ando A (2017). Impact of mild to moderate COPD on feasibility and prognosis in non-small cell lung cancer patients who received chemotherapy. Int J Chron Obstruct Pulmon Dis.

[32] Dubinett SM, Spira AE (2018). Impact of chronic obstructive pulmonary disease on immune-based treatment for lung cancer. Moving toward disease interception. Am J Respir Crit Care Med.

[33] Kobliakov VA (2010). Mechanisms of tumor promotion by reactive oxygen species. Biochemistry Biokhimiia.

[34] Sohal SS, Walters EH (2017). Essential need for rethink of COPD airway pathology: implications for new drug approaches for prevention of lung cancer as well as small airway fibrosis. Int J Chron Obstruct Pulmon Dis.

[35] Sohal SS, Walters EH (2013). Role of epithelial mesenchymal transition (EMT) in chronic obstructive pulmonary disease (COPD). Respir Res.

[36] Strzelak Agnieszka, Ratajczak Aleksandra, Adamiec Aleksander, Feleszko Wojciech (2018). Tobacco Smoke Induces and Alters Immune Responses in the Lung Triggering Inflammation, Allergy, Asthma and Other Lung Diseases: A Mechanistic Review. International Journal of Environmental Research and Public Health.

[37] Smith CJ, Hansch C (2000). The relative toxicity of compounds in mainstream cigarette smoke condensate. Food Chem Toxicol.

[38] Jemal A, Center MM, Ward E, Thun MJ (2009). Cancer occurrence. Method Mol Biol (Clifton, NJ).

[39] Maeda R, Tomita M, Usuda K, Uramoto H (2019). Clinicopathologic characteristics of non-small cell lung cancer in patients with smoking-related chronic obstructive pulmonary disease. Gen Thorac Cardiovasc Surg.

[40] Korpershoek YJ, Bruins Slot JC, Effing TW, Schuurmans MJ, Trappenburg JC (2017). Self-management behaviors to reduce exacerbation impact in COPD patients: a Delphi study. Int J Chron Obstruct Pulmon Dis.

[41] Sawa K, Koh Y, Kawaguchi T, Kambayashi S, Asai K, Mitsuoka S (2017). PIK3CA mutation as a distinctive genetic feature of non-small cell lung cancer with chronic obstructive pulmonary disease: a comprehensive mutational analysis from a multi-institutional cohort. Lung Cancer (Amsterdam, Netherlands).

[42] Zhang X, Jiang N, Wang L, Liu H, He R (2017). Chronic obstructive pulmonary disease and risk of lung cancer: a meta-analysis of prospective cohort studies. Oncotarget.

[43] Sundar IK, Mullapudi N, Yao H, Spivack SD, Rahman I (2011). Lung cancer and its association with chronic obstructive pulmonary disease: update on nexus of epigenetics. Curr Opin Pulm Med.

[44] Durham AL, Adcock IM (2015). The relationship between COPD and lung cancer. Lung Cancer (Amsterdam, Netherlands).

[45] Ng Kee Kwong Francois, Nicholson Andrew G., Harrison Celeste L., Hansbro Philip M., Adcock Ian M., Chung Kian Fan (2017). Is mitochondrial dysfunction a driving mechanism linking COPD to nonsmall cell lung carcinoma?. European Respiratory Review.

[46] Sanchez-Salcedo P, Zulueta JJ (2016). Lung cancer in chronic obstructive pulmonary disease patients, it is not just the cigarette smoke. Curr Opin Pulm Med.

[47] Zuo L, He F, Sergakis GG, Koozehchian MS, Stimpfl JN, Rong Y (2014). Interrelated role of cigarette smoking, oxidative stress, and immune response in COPD and corresponding treatments. Am J Physiol Lung Cell Mol Physiol.

[48] Perret JL, Walters EH, Abramson MJ, McDonald CF, Dharmage SC (2014). The independent and combined effects of lifetime smoke exposures and asthma as they relate to COPD. Expert Rev Respir Med.

[49] Churg A, Marshall CV, Sin DD, Bolton S, Zhou S, Thain K (2012). Late intervention with a myeloperoxidase inhibitor stops progression of experimental chronic obstructive pulmonary disease. Am J Respir Crit Care Med.

[50] Arnson Y, Shoenfeld Y, Amital H (2010). Effects of tobacco smoke on immunity, inflammation and autoimmunity. J Autoimmun.

[51] Sun T, Du W, Xiong H, Yu Y, Weng Y, Ren L (2014). TMEFF2 deregulation contributes to gastric carcinogenesis and indicates poor survival outcome. Clin Cancer Res.

[52] Schmidt N, Peitz U, Lippert H, Malfertheiner P (2005). Missing gastric cancer in dyspepsia. Aliment Pharmacol Ther.

[53] Coleman MP, Gatta G, Verdecchia A, Esteve J, Sant M, Storm H (2003). EUROCARE-3 summary: cancer survival in Europe at the end of the 20th century. Ann Oncol.

[54] Del Moral-Hernandez O, Castanon-Sanchez CA, Reyes-Navarrete S, Martinez-Carrillo DN, Betancourt-Linares R, Jimenez-Wences H (2019). Multiple infections by EBV, HCMV and Helicobacter pylori are highly frequent in patients with chronic gastritis and gastric cancer from Southwest Mexico: an observational study. Medicine..

[55] Tirado-Hurtado I, Carlos C, Lancho L, Alfaro A, Ponce R, Schwarz LJ (2019). Helicobacter pylori: history and facts in Peru. Crit Rev Oncol Hematol.

[56] Wessler S, Krisch LM, Elmer DP, Aberger F (2017). From inflammation to gastric cancer—the importance of Hedgehog/GLI signaling in Helicobacter pylori-induced chronic inflammatory and neoplastic diseases. Cell Communication and Signaling: CCS.

[57] Kamangar F, Sheikhattari P, Mohebtash M (2011). Helicobacter pylori and its effects on human health and disease. Arch Iran Med.

[58] Sanders MK, Peura DA (2002). Helicobacter pylori-associated diseases. Curr Gastroenterol Rep.

[59] Sjomina Olga, Heluwaert Frederic, Moussata Driffa, Leja Marcis (2017). Helicobacter pyloriinfection and nonmalignant diseases. Helicobacter.

[60] Pinto-Santini D, Salama NR (2005). The biology of Helicobacter pylori infection, a major risk factor for gastric adenocarcinoma. Cancer Epidemiol Biomarkers Prev.

[61] Uemura N, Okamoto S, Yamamoto S, Matsumura N, Yamaguchi S, Yamakido M (2001). Helicobacter pylori infection and the development of gastric cancer. N Engl J Med.

[62] Subhash VV, Ho B (2015). Inflammation and proliferation—a causal event of host response to Helicobacter pylori infection. Microbiology (Reading, England).

[63] Qadri Q, Rasool R, Gulzar GM, Naqash S, Shah ZA (2014). H. pylori infection, inflammation and gastric cancer. J Gastrointest Cancer.

[64] Bornschein J, Kandulski A, Selgrad M, Malfertheiner P (2010). From gastric inflammation to gastric cancer. Dig Dis (Basel, Switzerland).

[65] Noto JM, Peek RM (2011). The role of microRNAs in Helicobacter pylori pathogenesis and gastric carcinogenesis. Front Cell Infect Microbiol.

[66] Peek RM, Crabtree JE (2006). Helicobacter infection and gastric neoplasia. J Pathol.

[67] Naito Y, Yoshikawa T (2002). Molecular and cellular mechanisms involved in Helicobacter pylori-induced inflammation and oxidative stress. Free Radic Biol Med.

[68] Tsutsumi R, Higashi H, Higuchi M, Okada M, Hatakeyama M (2003). Attenuation of Helicobacter pylori CagA x SHP-2 signaling by interaction between CagA and C-terminal Src kinase. J Biol Chem.

[69] Hatakeyama M (2004). Oncogenic mechanisms of the Helicobacter pylori CagA protein. Nat Rev Cancer.

[70] Tatsuta M, Iishi H, Nakaizumi A, Okuda S, Taniguchi H, Hiyama T (1993). Fundal atrophic gastritis as a risk factor for gastric cancer. Int J Cancer.

[71] Sipponen P, Kekki M, Haapakoski J, Ihamaki T, Siurala M (1985). Gastric cancer risk in chronic atrophic gastritis: statistical calculations of cross-sectional data. Int J Cancer.

[72] Kinoshita Hiroto, Hayakawa Yoku, Koike Kazuhiko (2017). Metaplasia in the Stomach—Precursor of Gastric Cancer?. International Journal of Molecular Sciences.

[73] Chung HW, Lim JB (2014). Role of the tumor microenvironment in the pathogenesis of gastric carcinoma. World J Gastroenterol.

[74] Segal ED, Lange C, Covacci A, Tompkins LS, Falkow S (1997). Induction of host signal transduction pathways by Helicobacter pylori. Proc Natl Acad Sci U S A.

[75] Hollenbach Marcus (2017). The Role of Glyoxalase-I (Glo-I), Advanced Glycation Endproducts (AGEs), and Their Receptor (RAGE) in Chronic Liver Disease and Hepatocellular Carcinoma (HCC). International Journal of Molecular Sciences.

[76] Sepehri Z, Kiani Z, Kohan F, Alavian SM, Ghavami S (2017). Toll like receptor 4 and hepatocellular carcinoma; a systematic review. Life Sci.

[77] Lu WQ, Qiu JL, Huang ZL, Liu HY (2016). Enhanced circulating transforming growth factor beta 1 is causally associated with an increased risk of hepatocellular carcinoma: a Mendelian randomization meta-analysis. Oncotarget..

[78] Takeda H, Takai A, Inuzuka T, Marusawa H (2017). Genetic basis of hepatitis virus-associated hepatocellular carcinoma: linkage between infection, inflammation, and tumorigenesis. J Gastroenterol.

[79] Sekiba K, Otsuka M, Ohno M, Yamagami M, Kishikawa T, Suzuki T (2018). Hepatitis B virus pathogenesis: fresh insights into hepatitis B virus RNA. World J Gastroenterol.

[80] Xie M, Yang Z, Liu Y, Zheng M (2018). The role of HBV-induced autophagy in HBV replication and HBV related-HCC. Life Sci.

[81] Gómez-Moreno Andoni, Garaigorta Urtzi (2017). Hepatitis B Virus and DNA Damage Response: Interactions and Consequences for the Infection. Viruses.

[82] Polaris Observatory Collaborators (2018). Global prevalence, treatment, and prevention of hepatitis B virus infection in 2016: a modelling study. Lancet Gastroenterol Hepatol.

[83] Ringehan Marc, McKeating Jane A., Protzer Ulrike (2017). Viral hepatitis and liver cancer. Philosophical Transactions of the Royal Society B: Biological Sciences.

[84] Yang R, Xu Y, Dai Z, Lin X, Wang H (2018). The immunologic role of gut microbiota in patients with chronic HBV infection. J Immunol Res.

[85] Xie Y (2017). Hepatitis B virus-associated hepatocellular carcinoma. Adv Exp Med Biol.

[86] Ganem D, Prince AM (2004). Hepatitis B virus infection—natural history and clinical consequences. N Engl J Med.

[87] Xu W, Yu J, Wong VW (2017). Mechanism and prediction of HCC development in HBV infection. Best Pract Res Clin Gastroenterol.

[88] He G, Karin M (2011). NF-kappaB and STAT3—key players in liver inflammation and cancer. Cell Res.

[89] Tu T, Buhler S, Bartenschlager R (2017). Chronic viral hepatitis and its association with liver cancer. Biol Chem.

[90] Ivanov AV, Bartosch B, Smirnova OA, Isaguliants MG, Kochetkov SN (2013). HCV and oxidative stress in the liver. Viruses..

[91] Nishikawa H, Osaki Y (2015). Liver cirrhosis: evaluation, nutritional status, and prognosis. Mediat Inflamm.

[92] Li H, Huang MH, Jiang JD, Peng ZG (2018). Hepatitis C: from inflammatory pathogenesis to anti-inflammatory/hepatoprotective therapy. World J Gastroenterol.

[93] Trivedi Sunny, Starz-Gaiano Michelle (2018). Drosophila Jak/STAT Signaling: Regulation and Relevance in Human Cancer and Metastasis. International Journal of Molecular Sciences.

[94] Zhou C, Chen X, Zeng W, Peng C, Huang G, Li X (2016). Propranolol induced G0/G1/S phase arrest and apoptosis in melanoma cells via AKT/MAPK pathway. Oncotarget..

[95] Liang H, Zheng QL, Fang P, Zhang J, Zhang T, Liu W (2017). Targeting the PI3K/AKT pathway via GLI1 inhibition enhanced the drug sensitivity of acute myeloid leukemia cells. Sci Rep.

[96] Zhou TY, Zhou YL, Qian MJ, Fang YZ, Ye S, Xin WX (2018). Interleukin-6 induced by YAP in hepatocellular carcinoma cells recruits tumor-associated macrophages. J Pharmacol Sci.

[97] Moussa MM, Helal NS, Youssef MM (2018). Significance of pSmad2/3 and Smad4 in hepatitis C virus-related liver fibrosis and hepatocellular carcinoma. APMIS.

[98] Kao CC, Yi G, Huang HC (2016). The core of hepatitis C virus pathogenesis. Curr Opin Virol.

[99] Vescovo T, Refolo G, Vitagliano G, Fimia GM, Piacentini M (2016). Molecular mechanisms of hepatitis C virus-induced hepatocellular carcinoma. Clin Microbiol Infect.

[100] Levrero M (2006). Viral hepatitis and liver cancer: the case of hepatitis C. Oncogene..

[101] Erhardt A, Hassan M, Heintges T, Haussinger D (2002). Hepatitis C virus core protein induces cell proliferation and activates ERK, JNK, and p38 MAP kinases together with the MAP kinase phosphatase MKP-1 in a HepG2 Tet-Off cell line. Virology..

[102] Mahmoudvand S, Shokri S, Taherkhani R, Farshadpour F (2019). Hepatitis C virus core protein modulates several signaling pathways involved in hepatocellular carcinoma. World J Gastroenterol.

[103] Ivanov AV, Smirnova OA, Petrushanko IY, Ivanova ON, Karpenko IL, Alekseeva E (2015). HCV core protein uses multiple mechanisms to induce oxidative stress in human hepatoma Huh7 cells. Viruses..

[104] Wang Z, Li Z, Ye Y, Xie L, Li W (2016). Oxidative stress and liver cancer: etiology and therapeutic targets. Oxidative Med Cell Longev.

[105] Quintanilla Miguel, Montero-Montero Lucía, Renart Jaime, Martín-Villar Ester (2019). Podoplanin in Inflammation and Cancer. International Journal of Molecular Sciences.

[106] Lee SJ, Kim J, Ko J, Lee EJ, Koh HJ, Yoon JS (2018). Tumor necrosis factor-like weak inducer of apoptosis induces inflammation in Graves’ orbital fibroblasts. PLoS One.

[107] Khodabandehlou N, Mostafaei S, Etemadi A, Ghasemi A, Payandeh M, Hadifar S (2019). Human papilloma virus and breast cancer: the role of inflammation and viral expressed proteins. BMC Cancer.

[108] Dinarello CA (2014). An expanding role for interleukin-1 blockade from gout to cancer. Mol Med (Cambridge, Mass).

[109] Garlanda C, Dinarello CA, Mantovani A (2013). The interleukin-1 family: back to the future. Immunity..

[110] Bent Rebekka, Moll Lorna, Grabbe Stephan, Bros Matthias (2018). Interleukin-1 Beta—A Friend or Foe in Malignancies?. International Journal of Molecular Sciences.

[111] Malik A, Kanneganti TD (2018). Function and regulation of IL-1alpha in inflammatory diseases and cancer. Immunol Rev.

[112] Carmi Y, Dotan S, Rider P, Kaplanov I, White MR, Baron R (2013). The role of IL-1beta in the early tumor cell-induced angiogenic response. J Immunol (Baltimore, Md : 1950).

[113] Saijo Y, Tanaka M, Miki M, Usui K, Suzuki T, Maemondo M (2002). Proinflammatory cytokine IL-1 beta promotes tumor growth of Lewis lung carcinoma by induction of angiogenic factors: in vivo analysis of tumor-stromal interaction. J Immunol (Baltimore, Md : 1950).

[114] Voronov E, Shouval DS, Krelin Y, Cagnano E, Benharroch D, Iwakura Y (2003). IL-1 is required for tumor invasiveness and angiogenesis. Proc Natl Acad Sci U S A.

[115] Tu S, Bhagat G, Cui G, Takaishi S, Kurt-Jones EA, Rickman B (2008). Overexpression of interleukin-1beta induces gastric inflammation and cancer and mobilizes myeloid-derived suppressor cells in mice. Cancer Cell.

[116] Tulotta C, Ottewell P (2018). The role of IL-1B in breast cancer bone metastasis. Endocr Relat Cancer.

[117] Apte RN, Krelin Y, Song X, Dotan S, Recih E, Elkabets M (2006). Effects of micro-environment- and malignant cell-derived interleukin-1 in carcinogenesis, tumour invasiveness and tumour-host interactions. Eur J Cancer (Oxford, England : 1990).

[118] Zhou W, Guo S, Gonzalez-Perez RR (2011). Leptin pro-angiogenic signature in breast cancer is linked to IL-1 signalling. Br J Cancer.

[119] Vidal-Vanaclocha F, Fantuzzi G, Mendoza L, Fuentes AM, Anasagasti MJ, Martin J (2000). IL-18 regulates IL-1beta-dependent hepatic melanoma metastasis via vascular cell adhesion molecule-1. Proc Natl Acad Sci U S A.

[120] Han TS, Voon DC, Oshima H, Nakayama M, Echizen K, Sakai E (2019). Interleukin 1 up-regulates microRNA 135b to promote inflammation-associated gastric carcinogenesis in mice. Gastroenterology.

[121] Huang FY, Chan AO, Rashid A, Wong DK, Seto WK, Cho CH (2016). Interleukin-1beta increases the risk of gastric cancer through induction of aberrant DNA methylation in a mouse model. Oncol Lett.

[122] Shigematsu Y, Niwa T, Rehnberg E, Toyoda T, Yoshida S, Mori A (2013). Interleukin-1beta induced by Helicobacter pylori infection enhances mouse gastric carcinogenesis. Cancer Lett.

[123] Voronov E, Dinarello CA, Apte RN (2018). Interleukin-1alpha as an intracellular alarmin in cancer biology. Semin Immunol.

[124] Kaminska K, Czarnecka AM, Escudier B, Lian F, Szczylik C (2015). Interleukin-6 as an emerging regulator of renal cell cancer. Urol Oncol.

[125] Francescone R, Hou V, Grivennikov SI (2015). Cytokines, IBD, and colitis-associated cancer. Inflamm Bowel Dis.

[126] Song Z, Ren D, Xu X, Wang Y (2018). Molecular cross-talk of IL-6 in tumors and new progress in combined therapy. Thorac Cancer.

[127] Tsukamoto H, Fujieda K, Senju S, Ikeda T, Oshiumi H, Nishimura Y (2018). Immune-suppressive effects of interleukin-6 on T-cell-mediated anti-tumor immunity. Cancer Sci.

[128] Rath T, Billmeier U, Waldner MJ, Atreya R, Neurath MF (2015). From physiology to disease and targeted therapy: interleukin-6 in inflammation and inflammation-associated carcinogenesis. Arch Toxicol.

[129] Unver N, McAllister F (2018). IL-6 family cytokines: key inflammatory mediators as biomarkers and potential therapeutic targets. Cytokine Growth Factor Rev.

[130] Kitamura H, Ohno Y, Toyoshima Y, Ohtake J, Homma S, Kawamura H (2017). Interleukin-6/STAT3 signaling as a promising target to improve the efficacy of cancer immunotherapy. Cancer Sci.

[131] Masjedi A, Hashemi V, Hojjat-Farsangi M, Ghalamfarsa G, Azizi G, Yousefi M (2018). The significant role of interleukin-6 and its signaling pathway in the immunopathogenesis and treatment of breast cancer. Biomed Pharmacother.

[132] Xu J, Ye Y, Zhang H, Szmitkowski M, Makinen MJ, Li P (2016). Diagnostic and prognostic value of serum interleukin-6 in colorectal cancer. Medicine..

[133] Kosmopoulos M, Christofides A, Drekolias D, Zavras PD, Gargalionis AN, Piperi C (2018). Critical role of IL-8 targeting in gliomas. Curr Med Chem.

[134] Yung MM, Tang HW, Cai PC, Leung TH, Ngu SF, Chan KK (2018). GRO-alpha and IL-8 enhance ovarian cancer metastatic potential via the CXCR2-mediated TAK1/NFkappaB signaling cascade. Theranostics..

[135] Sharma I, Singh A, Siraj F, Saxena S (2018). IL-8/CXCR1/2 signalling promotes tumor cell proliferation, invasion and vascular mimicry in glioblastoma. J Biomed Sci.

[136] Zheng Tingjin, Ma Guoxing, Tang Mingqing, Li Zhongwan, Xu Ruian (2018). IL-8 Secreted from M2 Macrophages Promoted Prostate Tumorigenesis via STAT3/MALAT1 Pathway. International Journal of Molecular Sciences.

[137] Fu S, Chen X, Lin HJ, Lin J (2018). Inhibition of interleukin 8/CX-C chemokine receptor 1,/2 signaling reduces malignant features in human pancreatic cancer cells. Int J Oncol.

[138] Shimizu M, Tanaka N (2019). IL-8-induced O-GlcNAc modification via GLUT3 and GFAT regulates cancer stem cell-like properties in colon and lung cancer cells. Oncogene..

[139] Fei M, Guan J, Xue T, Qin L, Tang C, Cui G (2018). Hypoxia promotes the migration and invasion of human hepatocarcinoma cells through the HIF-1alpha-IL-8-Akt axis. Cell Mol Biol Lett.

[140] Ning Y, Feng W, Cao X, Ren K, Quan M, Chen A (2019). Genistein inhibits stemness of SKOV3 cells induced by macrophages co-cultured with ovarian cancer stem-like cells through IL-8/STAT3 axis. J Exp Clin Cancer Res.

[141] Kawano M, Tanaka K, Itonaga I, Iwasaki T, Tsumura H (2018). Interaction between human osteosarcoma and mesenchymal stem cells via an interleukin-8 signaling loop in the tumor microenvironment. Cell Commun Signal.

[142] Esquivel-Velazquez M, Ostoa-Saloma P, Palacios-Arreola MI, Nava-Castro KE, Castro JI, Morales-Montor J (2015). The role of cytokines in breast cancer development and progression. J Interf Cytokine Res.

[143] Rossi S, Cordella M, Tabolacci C, Nassa G, D’Arcangelo D, Senatore C (2018). TNF-alpha and metalloproteases as key players in melanoma cells aggressiveness. J Exp Clin Cancer Res.

[144] Tan W, Luo X, Li W, Zhong J, Cao J, Zhu S (2019). TNF-alpha is a potential therapeutic target to overcome sorafenib resistance in hepatocellular carcinoma. EBioMedicine..

[145] Karin M, Greten FR (2005). NF-kappaB: linking inflammation and immunity to cancer development and progression. Nat Rev Immunol.

[146] de Visser KE, Coussens LM (2006). The inflammatory tumor microenvironment and its impact on cancer development. Contrib Microbiol.

[147] Wang J, Yao Y, Zhang Q, Li S, Tang L (2019). Inflammatory responses induced by Helicobacter pylori on the carcinogenesis of gastric epithelial GES1 cells. Int J Oncol.

[148] Li J, Lau G, Chen L, Yuan YF, Huang J, Luk JM (2012). Interleukin 23 promotes hepatocellular carcinoma metastasis via NF-kappa B induced matrix metalloproteinase 9 expression. PLoS One.

[149] Yang SM, Li SY, Hao-Bin Y, Lin-Yan X, Sheng X (2019). IL-11 activated by lnc-ATB promotes cell proliferation and invasion in esophageal squamous cell cancer. Biomed Pharmacother.

[150] Lay V, Yap J, Sonderegger S, Dimitriadis E (2012). Interleukin 11 regulates endometrial cancer cell adhesion and migration via STAT3. Int J Oncol.

[151] Wang R, Lou X, Feng G, Chen J, Zhu L, Liu X (2019). IL-17A-stimulated endothelial fatty acid beta-oxidation promotes tumor angiogenesis. Life Sci.

[152] Lv Q, Wu K, Liu F, Wu W, Chen Y, Zhang W (2018). Interleukin17A and heparanase promote angiogenesis and cell proliferation and invasion in cervical cancer. Int J Oncol.

[153] Zaynagetdinov R, Sherrill TP, Gleaves LA, McLoed AG, Saxon JA, Habermann AC (2015). Interleukin-5 facilitates lung metastasis by modulating the immune microenvironment. Cancer Res.

[154] Lee EJ, Lee SJ, Kim S, Cho SC, Choi YH, Kim WJ (2013). Interleukin-5 enhances the migration and invasion of bladder cancer cells via ERK1/2-mediated MMP-9/NF-kappaB/AP-1 pathway: involvement of the p21WAF1 expression. Cell Signal.

[155] Zhang JF, Wang P, Yan YJ, Li Y, Guan MW, Yu JJ (2017). IL33 enhances glioma cell migration and invasion by upregulation of MMP2 and MMP9 via the ST2-NF-kappaB pathway. Oncol Rep.

[156] Li Y, Shi J, Qi S, Zhang J, Peng D, Chen Z (2018). IL-33 facilitates proliferation of colorectal cancer dependent on COX2/PGE2. J Exp Clin Cancer Res.

[157] Liu X, Hansen DM, Timko NJ, Zhu Z, Ames A, Qin C (2019). Association between interleukin33 and ovarian cancer. Oncol Rep.

[158] Gupta R, Yan XJ, Barrientos J, Kolitz JE, Allen SL, Rai K (2018). Mechanistic insights into CpG DNA and IL-15 synergy in promoting B cell chronic lymphocytic leukemia clonal expansion. J Immunol (Baltimore, Md : 1950).

[159] Levina V, Nolen BM, Marrangoni AM, Cheng P, Marks JR, Szczepanski MJ (2009). Role of eotaxin-1 signaling in ovarian cancer. Clin Cancer Res.

[160] Tian M, Chen L, Ma L, Wang D, Shao B, Wu J (2016). Expression and prognostic significance of CCL11/CCR3 in glioblastoma. Oncotarget.

[161] Zhang J, Chen Y, Chen K, Huang Y, Xu X, Chen Q (2019). IL-33 drives the antitumour effects of dendritic cells via upregulating CYLD expression in pulmonary adenocarcinoma. Artificial Cells, Nanomedicine, and Biotechnology.

[162] Cano Sanchez Mariola, Lancel Steve, Boulanger Eric, Neviere Remi (2018). Targeting Oxidative Stress and Mitochondrial Dysfunction in the Treatment of Impaired Wound Healing: A Systematic Review. Antioxidants.

[163] Avishai E, Yeghiazaryan K, Golubnitschaja O (2017). Impaired wound healing: facts and hypotheses for multi-professional considerations in predictive, preventive and personalised medicine. EPMA J.

[164] Stolzenburg-Veeser L, Golubnitschaja O (2018). Mini-encyclopaedia of the wound healing—opportunities for integrating multi-omic approaches into medical practice. J Proteome.

[165] Golubnitschaja O, Flammer J (2018). Individualised patient profile: clinical utility of Flammer syndrome phenotype and general lessons for predictive, preventive and personalised medicine. EPMA J.

[166] Roos A, Byron SA (2019). Genomics-enabled precision medicine for cancer. Cancer Treat Res.

[167] Qian S, Yang Y, Li N, Cheng T, Wang X, Liu J (2018). Prolactin variants in human pituitaries and pituitary adenomas identified with two-dimensional gel electrophoresis and mass spectrometry. Front Endocrinol.

[168] Zhan X, Yang H, Peng F, Li J, Mu Y, Long Y (2018). How many proteins can be identified in a 2DE gel spot within an analysis of a complex human cancer tissue proteome?. Electrophoresis..

[169] Zhan X, Long Y, Lu M (2018). Exploration of variations in proteome and metabolome for predictive diagnostics and personalized treatment algorithms: innovative approach and examples for potential clinical application. J Proteome.

[170] Kunin A, Polivka J, Moiseeva N, Golubnitschaja O (2018). “Dry mouth” and “Flammer” syndromes—neglected risks in adolescents and new concepts by predictive, preventive and personalised approach. EPMA J.

[171] Goncharenko V, Bubnov R, Polivka J, Zubor P, Biringer K, Bielik T (2019). Vaginal dryness: individualised patient profiles, risks and mitigating measures. EPMA J.

[172] Latifi Rifat (2019). The Modern Hospital.

[173] Golubnitschaja Olga (2019). Flammer Syndrome.

[174] Bubnov R, Polivka J, Zubor P, Konieczka K, Golubnitschaja O (2017). “Pre-metastatic niches” in breast cancer: are they created by or prior to the tumour onset? “Flammer syndrome” relevance to address the question. EPMA J.

